# Case Report: A case report highlighting bilateral EDB wasting as a clinical marker for lumbar canal stenosis

**DOI:** 10.12688/f1000research.6865.1

**Published:** 2015-08-04

**Authors:** Bijoy Mohan Kumar, Sunil Munakomi

**Affiliations:** 1 Department of Neurosurgery, College of Medical Sciences, Chitwan, Nepal

**Keywords:** extensor digitorum brevis, lumbar canal stenosis, dural wasting

## Abstract

Herein we discuss a case of a 55 year old male presenting with history suggestive of sciatica on the left leg. Straight leg raising (SLR) test was positive at 45 degrees on the left side. His ankle reflex was absent and the power of extensor hallusus longus (EHL) was 4/5 on the same side. MRI lumbosacral spine revealed left paramedian disc prolapsed on L4/L5 level with spinal canal diameter of 9mm.However since his bilateral extensor digitorm brevis (EDB) were wasted, we suspected associated lumbar canal stenosis and thereby opted for laminectomy and discectomy in this case. Intraoperatively dural wasting, hypertrophied facets and narrow canal were confirmed. Laminectomy, medial facectectomy and discectomy were carried out. The patient recovered uneventfully with resolution of his sciatica-like pain. Bilateral EDB wasting thereby provides a clinical clue to the underlying lumbar canal stenosis and can help in making correct therapeutic decisions.

## Introduction

Lumbar disc herniation mostly causes radicular symptoms but can also lead to lumbar canal stenosis ^1, 2^. Tackling only the disc may not suffice in improving the symptomatology in patients and can invariably lead to failed back syndrome. Wasting of the extensor digitorum brevis (EDB) has been previously used as a marker for L5/S1 radiculopathy ^3, 4^. Herein we highlight the clinical importance of observing for evidence of bilateral EDB wasting as a marker for underlying lumbar canal stenosis. This simple clinical observation can help decide the correct surgical strategy and thereby prevent failed back syndrome by carrying out laminectomy rather than just tackling the disc by performing minimally invasive discectomy.

## Case report

**Figure 1.**
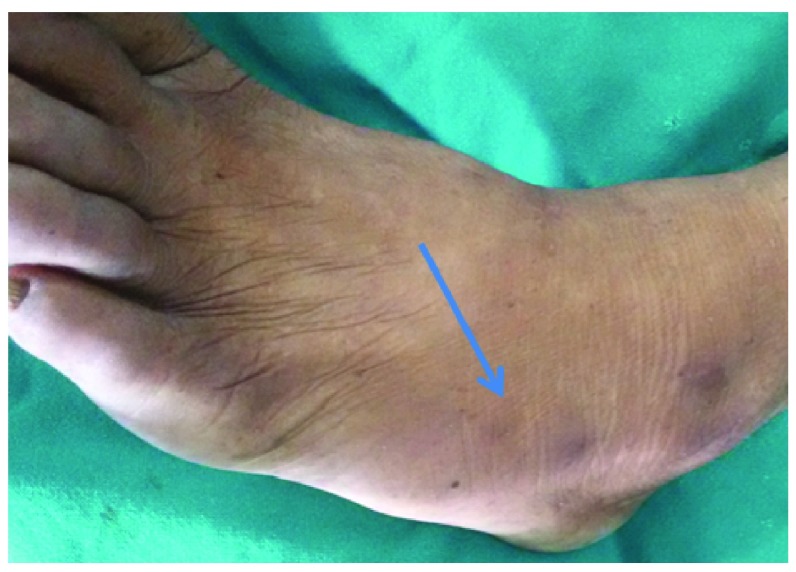
Extensor digitorum brevis (EDB) wasting (arrow head) in a patient with lumbar canal stenosis.

**Figure 2.**
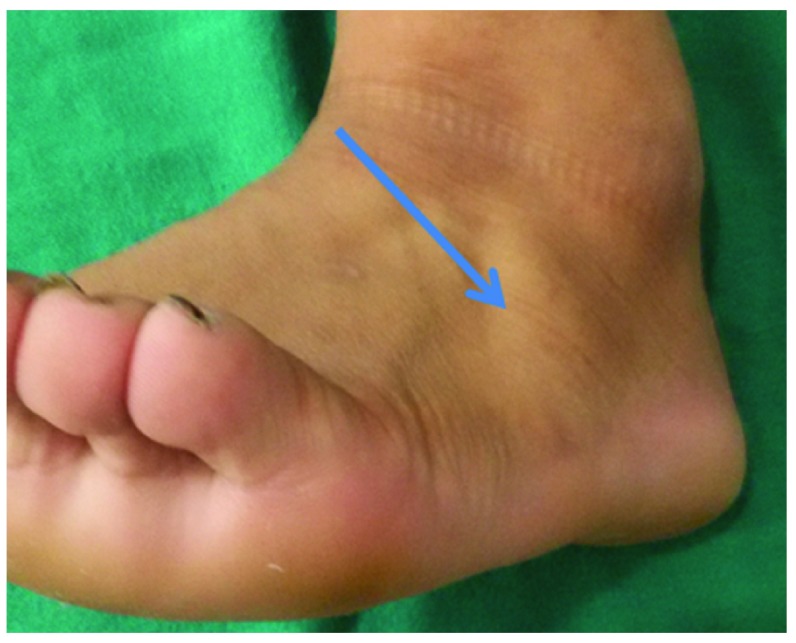
Normal EDB (arrow head) in a healthy volunteer.

**Figure 3.**
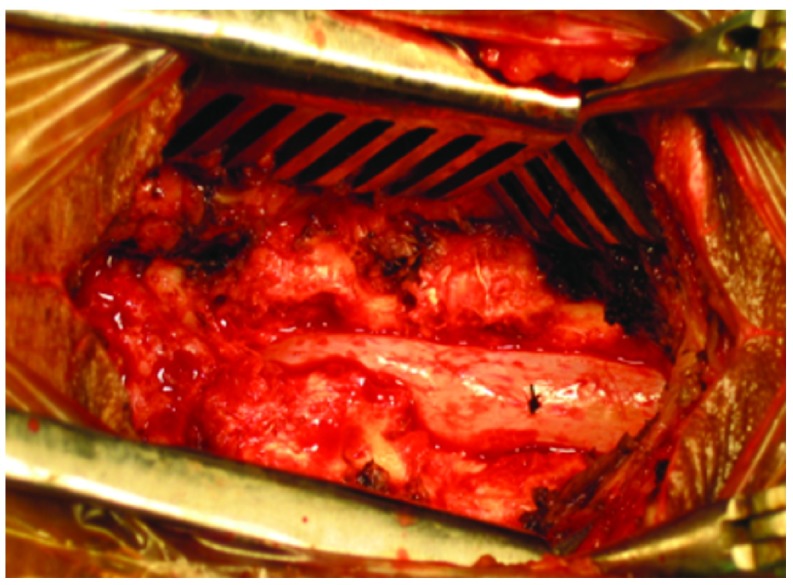
Intraoperative dural compression and wasting seen in a case of canal stenosis.

**Figure 4.**
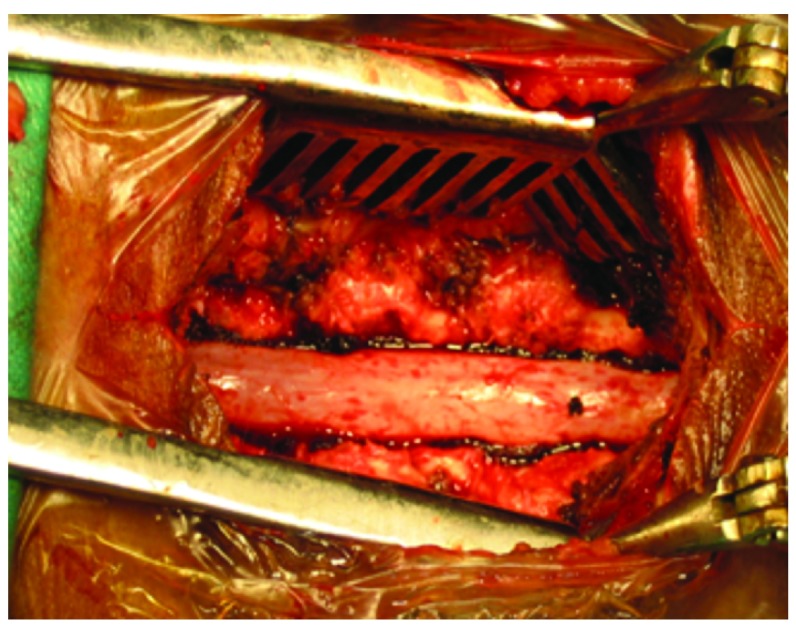
Lax dura seen after decompressive laminectomy.

A 55 year old male from Lumbini, Nepal presented to us with a history of low back pain for 4 months with recent onset sciatica on his left side. There was no history suggestive of vascular claudication. His bladder and bowel habit was normal. His peripheral pulses in the legs were normal. There was no significant past medical or surgical illnesses. The patient had been taking oral analgesics for his pain that reduced his pain to some extent. Examination revealed straight leg raising (SLR) of 45 degrees on his left leg. Left ankle reflex was absent. The power of the extensor hallusus longus (EHL) on his left leg was 4/5. Pain sensation was diminished on his left first dorsal web space and the lateral part of the foot dorsum. However his bilateral EDB muscles were wasted ( Figure 1, Figure 2) and so, clinical diagnosis of L4/L5 disc with canal stenosis was made. MRI lumbar spine revealed L4/L5 left paramedian disc with a canal diameter of 9mm. Dynamic X-ray of the lumbar spine did not show any instability. Because of the presence of bilateral EDB wasting, we opted for laminectomy in the patient rather than minimal access discectomy. Removing only the disc might result in failed back syndrome in such a patient. After detailed counseling regarding the disease process, probable complications, benefits and risks of different modes of surgical management and obtaining both written and verbal consent from the patient’s son akin, we posted the case for surgery. Intraoperatively, hypertrophic facet joints and a narrow canal were confirmed. There was significant dural wasting ( Figure 3). We performed discectomy, bilateral medial facetectomy and laminectomy on the corresponding level ( Figure 4). Postoperative there was resolution of the sciatica-like pain and the patient was mobilized from the second postoperative day. The patient was started on tablet pregabalin 75 mg and tablet methycobalamine 1500 µg once daily orally for 3 weeks. Patient follow-up one month later revealed no new symptoms. The patient was advised to perform regular back exercises and physiotherapy. Dynamic lumbar spine X-ray did not reveal any instability.

## Discussion

With the increasing longevity and continually climbing proportion of middle-aged and elderly persons, low back ache is surely going to be a ubiquitous and disabling disease of mankind ^2^.

The diagnosis of spinal stenosis is normally aided by radiological studies ^5^. CT of the lumbar spine can show characteristic trefoil appearance of the canal while MRI can show loss of CSF surrounding the canal. However, in developing countries like ours, radiological studies may be limited due to a lack of patient finances and hospital resources. As a result, doctors are limited to clinical diagnosis.

Management of lumbar disc disease ranges from conservative ^6^ to epidural steroids injection ^7, 8^ and surgery ^9^. However, failure to correctly diagnose and treat canal stenosis may invariably lead to failed back syndrome in patients ^10^.

The role of EDB as a clinical indicator of the L5 radiculopathy has already been proven ^3, 4^. Therefore, simple assessment of the bulk of the EDB muscle on both sides can predict the underlying canal stenosis and thereafter help make correct therapeutic decisions.

In this era of minimally invasive procedures, this simple bedside marker provides a word of caution for novices in the vast realms of lumbar spine procedures.

## Conclusion

Bilateral EDB wasting can be taken as a reliable clinical marker for the diagnosis of lumbar canal stenosis. This simple bedside observation can aid us in deciding on the correct surgical strategy and thereby prevent failed back syndrome if we happen to miss the underlying canal stenosis and instead manage the disc only. It is a clinical pearl for general doctors working in remote areas to correctly assess and refer patients with EBD wasting to tertiary care centres from a subset of patients presenting with low back ache.

## Consent

Both written and verbal informed consent for publication of images and clinical data related to this case was sought and obtained from the son of the patient.
