# Supplementary material for: Case Report: A case report highlighting bilateral EDB wasting as a clinical marker for lumbar canal stenosis
Source: F1000Res. 2015 Aug 4;4:467. [Version 1] doi: 10.12688/f1000research.6865.1 (PMC4608354; doi:10.12688/f1000research.6865.1)
Supplement: Peer review history

# Peer review: Referee response for version 1

**DOI:** 10.5256/f1000research.7391.r10763

**Published:** 2015-10-12

**Authors:** Rully Hanafi Dahlan, Farid Yudoyono

**Affiliations:** 1 Department of Neurosurgery, Padjadjaran University, Bandung, Indonesia

I have following points that should be clearly described:

1. Fig 3 and 4 show surgical views. Authors must state with symbols (asterisks etc) to show what level of surgical work.
2. We would suggest a detailed axial and sagital view of the MRI for the detail level of lumbar canal compression.
3. EDB is sensitive for L5-S1 radiculopathy. Authors must verify it with EMG modality.

We have read this submission. We believe that we have an appropriate level of expertise to confirm that it is of an acceptable scientific standard.

# Peer review: Referee response for version 1

**DOI:** 10.5256/f1000research.7391.r10565

**Published:** 2015-09-28

**Authors:** Mehmet Zileli

**Affiliations:** 1 Department of Neurosurgery, Ege University, Izmir, Turkey

This report tries to show the importance of muscle atrophy of extensor hallusus (EHL) longus in diagnosis of lumbar spinal stenosis. I have following criticisms:

1. They must place MRI images of the patient instead of intraoperative surgical views.
2. They claim that atrophy of EHL on both sides shows the disease is spinal canal stenosis instead of disc herniation. However they do not have any evidence that it cannot be produced by a midline disc herniation. Nor can a case report make us reach a conclusion like that.
3. Atrophy of the EHL muscles should be justified by muscle strength measurements and EMG.

I have read this submission. I believe that I have an appropriate level of expertise to confirm that it is of an acceptable scientific standard, however I have significant reservations, as outlined above.

# Peer review: Referee response for version 1

**DOI:** 10.5256/f1000research.7391.r9970

**Published:** 2015-08-24

**Authors:** Amit Thapa

**Affiliations:** 1 Unit of Neurological Surgery, Kathmandu Medical College, Kathmandu, Nepal

The authors Kumar *et al. *in this article have persuasively highlighted the importance of clinical examination while deciding on a case of low back ache with radiculopathy. This is becoming ever vital as incidence of failed back syndrome is on a rise, a major causative factor being incomplete clinical evaluation of patient ^1^. However few points need to be highlighted.

1. Since the patient had been complaining of low back ache for 4 months and only recently started having sciatica, it would be prudent to ask patient regarding claudication and relation of position on pain. The first question would tell us about canal stenosis and the later about facet arthropathy. This is important in this case, as the procedure done on the patient would not relieve the facet arthropathy and patient would keep on complaining of axial back ache later.
2. As this case highlights co existence of degenerative lumbar spinal stenosis and disc herniation, more discussion on lumbar spinal stenosis is warranted. Degenerative lumbar spinal stenosis describes a condition in which there is diminished space available for the neural and vascular elements in the lumbar spine secondary to degenerative changes in the spinal canal, presenting with a history of gluteal or lower extremity symptoms exacerbated by walking or standing which improves or resolves with sitting or bending forward. Patients whose pain is not made worse with walking have a low likelihood of stenosis ^2^.
3. We would request more details on the MRI study if possible an axial film of the site of compression. Guideline suggest getting a thin (4-5 mm) MRI sections with a combination of T1, proton density and T2 pulse sequences in both axial and sagittal planes with additional angled and stacked axial sections ^2^. Meta-analysis has shown sensitivity of MRI in the diagnosis of adult spinal stenosis to be 81-97%, of CT 70-100% and myelography 67-78% ^3^. Besides the antero-posterior diameter (< 10 mm) and cross-sectional area (< 70 mm ^2^) of spinal canal,  MRI finding of positive sedimentation sign is a good positive sign to rule in lumbar spinal stenosis with high specificity and sensitivity ^4^.
4. Authors have correctly highlighted the importance of dynamic x ray study to see for any instability. In presence of instability based on Posner’s criteria, patient should be offered decompression with fusion if the stenosis is moderate to severe ^2^. However we stress on the need of standing full-length lateral radiographs of the spine to check for sagittal balance of the patient which has bearing of increasing instability after performing procedures like laminectomy. In particular, 3 measures are of vital importance: (1) global sagittal balance (C7 plumb line [C7PL], C7/sacro-femoral distance ratio, and spino-sacral angle), (2) spino-pelvic morphology (pelvic incidence, sacral slope, and pelvic tilt), and (3) spinal parameters (lumbar lordosis and thoracic kyphosis). Jeon *et al.* have found posterior migration of the C7PL and increase lumbar lordosis following decompressive laminectomy, in their evaluation of 40 patients over 2 years ^5^.
5. Figure 1 and 2 shows EDB located laterally than are expected. Authors must have drawn them to show the visible differences. However to see for EDB, best location would be interdigital spaces over dorsal of foot as EDB helps extend digits 2 though 4 (see figure) ^6^.
6. EDB being a muscle with smallest bulk in foot is clinically very sensitive for L5 radiculopahty. This is a case report, similar to earlier reports in cases of spina bifida or tethered cord syndrome where late manifestation has led to EDB weakness ^7^. However North American Spine Society (NASS) in their recommendation, have found insufficient evidence to make a recommendation for or against certain physical findings for the diagnosis of degenerative lumbar spinal stenosis including an abnormal Romberg test, thigh pain exacerbated with extension, sensorimotor deficits, leg cramps and abnormal Achilles tendon reflexes ^2^.

To conclude, the authors have genuinely stress on the need of comprehensive clinical evaluation of spine and neurological function before embarking on surgical management of low back ache or radiculopathy. This paper supports the case with a good summary.

I have read this submission. I believe that I have an appropriate level of expertise to confirm that it is of an acceptable scientific standard.

## References

[1] LongDM: Failed back surgery syndrome. *Neurosurg Clin N Am*.1991;2(4) :899-919 1840393

[2] Diagnosis and Treatment of Degenerative Lumbar Spinal Stenosis. *North American Spine Society*.2011; Reference source

[3] KentDLHaynorDRLarsonEBDeyoRA: Diagnosis of lumbar spinal stenosis in adults: a metaanalysis of the accuracy of CT, MR, and myelography. *AJR Am J Roentgenol*.1992;158(5) : 10.2214/ajr.158.5.1533084 1135-1144 10.2214/ajr.158.5.1533084 Reference source 1533084

[4] DawoodOMHassanTAMoheyN: The MRI finding of the nerve root sedimentation sign: Its clinical validity and operative relativity for patients with lumbar spinal stenosis. *The Egyptian Journal of Radiology and Nuclear Medicine*.2014;45: doi:10.1016/j.ejrnm.2013.11.011 203-209 10.1016/j.ejrnm.2013.11.011 Reference source

[5] JeonCHLeeHDLeeYSSeoHSChungNS: Change in sagittal profiles after decompressive laminectomy in patients with lumbar spinal canal stenosis: a 2-year preliminary report. *Spine (Phila Pa 1976)*.2015;40(5) : 10.1097/BRS.0000000000000745 E279-E285 10.1097/BRS.0000000000000745 25901983

[6] Dorsum of Foot. Deep dissection. *Wikimedia Commons*.2013; Reference source

[7] MacnicolMFNadeemRD: Evaluation of the deformity in club foot by somatosensory evoked potentials. *J Bone Joint Surg Br*.2000;82:731-735 Reference source 1096317510.1302/0301-620x.82b5.9988
